# Collapsing occupational identities on social media: new approaches to digital identity management

**DOI:** 10.1007/s44538-026-00004-x

**Published:** 2026-07-22

**Authors:** Carolina Are, Pamela Briggs

**Affiliations:** 1 LSE100, London School of Economics and Political Science, London, UK; 2 Department of Psychology, Northumbria University, Newcastle upon Tyne, UK

**Keywords:** Context collapse, Context collusion, Digital identity, Content creators, Human resources

## Abstract

This study observes social media users who have collapsed digital occupational identities in a professional landscape characterised both by precarity and by the need to find meaning at work. It aims to understand how and why individuals straddling strikingly different work identities decide to make both identities public, sharing context and/or content online in an intentional process described as ‘context collusion’. Towards this, we present five case studies and an autoethnographic conversation that show how the platformisation of working life, coupled with new SM affordances and expectations, can generate identity management challenges that go well beyond ideas of non-consensual or unintentional context collapse or the sanitisation of contrasting but simultaneous identities. These cases illustrate individuals from widely different domains, including some who hold and promote an identity in relation to sex-related work. We do this as a deliberate strategy to highlight similarities between conventional and sexual labour. We found that those who successfully joined their two work identities collapsed not just their contexts but their content as part of their self-presentation, sharing both aspects of their lives publicly but with distinct narratives behind their choice to do so. However, we found a distinct tension between traditional work processes and policies and the needs of those adopting content creation as a side hustle or as a means of promoting other work.

## Introduction

Our paper observes social media (SM) users holding multiple but distinctly different work identities, who have deliberately made both identities public on their SM platforms. We seek to understand how and why these individuals share strikingly different work contexts and/or content online in an intentional process described as ‘context collusion’ by Davis and Jurgenson (2014), also reflecting Duguay’s (2022) concept of identity modulation. We note the cognitive and emotional labour involved in moderating or managing different work identities, often driven by the need to find meaning at work and to establish the legitimacy of a second career. However, we also note the way that the Human Resources (HR) demands within the more ‘traditional’ workplace sometimes hindered those seeking to adopt content creation as a side hustle or as a means of promoting other work.

Through the lenses of platformisation, or “the penetration of the infrastructures, economic processes, and governmental frameworks of platforms in different economic sectors and spheres of life” (Poell et al., 2019:5), and influencer creep (Bishop, 2023), or the creeping of influencer practices into everyday life, we aim to elicit new ways of thinking about the ways we manage multiple, different versions of self at the intersection of digital labour and identity. To do so, we present five case studies and an autoethnographic conversation, arguing that these new demands born out of the need to constantly engage in SM promotion require new approaches to identity management research, moving beyond the view that individuals with strikingly different professional and/or personal identities will almost inevitably wish to keep these separate.

Until recently, the prevailing research assumption was that people sought to avoid ‘context collapse’, or situations where “people, information, and norms from different settings all converge into one context” (Loh & Walsh, 2021:1). Context collapse is a well-researched phenomenon, known to be exacerbated by SM’s lack of spatial, social or temporal boundaries (Kaul & Chaudri, 2018). In the work context, researchers typically focus on people’s need to retain a strong professional identity, untainted by life outside of the workplace, because *unintended* context collapse (e.g. following leaked SM posts) can limit employment and promotion opportunities, or even lead to job loss (Robards & Graf, 2022).

However, this view is becoming increasingly challenging at a time when SM have become corporate ecosystems (Poell, 2020) that not only offer content creation opportunities, but that also provide advertising, identity services, networking, software and hardware etc. People who engage in more than one type of work may therefore *willingly* choose to share information about their distinct identities, i.e. they may deliberately engage in *context collusion*, where they blur the boundaries of radically different and somewhat challenging selves (Davis & Jurgenson, 2014). This is the focus of our work, where we sought out individuals who hold two very different professional lives and who make the decision to make both of their working selves public, promoting highly different work identities across a range of contexts and platforms.

Essential to this work is the understanding that, for many individuals, success within a workplace now involves some degree of self-promotion. SM affect all aspects of our lives (Baym & boyd, 2012), and ‘influencer creep’ (Bishop, 2023) is almost taken for granted: we regularly broadcast information relevant to both home and work, and we have to engage in self-branding to consistently present a digital identity. This may involve simply sharing a new job opportunity, but it can go much further: professionals increasingly take on an ‘influencer’ role associated with their workplace to increase their employability or visibility in a precarious job market (ibid.).

This presents complex identity management challenges, as Atef et al. (2023) noted when interviewing doctors who also created and promoted their own health-related vlogs. These medical professionals were able to promote a relatively congruent core identity (the trusted physician). However, the blurred boundaries between medical professional and content creator meant they were constantly required to engage in ‘face work’ to impress both audiences. This is a prime example of the challenges and opportunities of context collusion, albeit featuring different professional identities that are somewhat congruent, and connected to medical expertise.

We instead sought to highlight the identity management challenges faced by people working two totally different, relatively incompatible jobs, but who nonetheless decided to make both working lives public, answering the following research questions:

### RQ1

Why do multifaceted social media users seek to willingly collapse their contexts?

### RQ2

What are the benefits of collapsing one’s social media context?

### RQ3

What are the challenges of collapsing one’s social media context?

We deliberately included cases where one of the professional identities was stigmatised. As a result, the five cases we present include three individuals who hold and promote one identity in relation to sex-related work, recognising that such people face additional challenges in a society that still fails to recognise sexual labour (Leigh, 1979).

With these five case studies, we illustrate some of the complexities of context collusion in a platformised society that rewards digital self-promotion in times of increasing precarity, highlighting the gains and losses that follow increased personal transparency, particularly in stigmatised contexts.

## Social media publicness and work

Sharing aspects of personal and/or work lives via SM can be traced back to the evolution of taking and sharing photographs (Vivienne & Burgess, 2013): what originated as an intimate practice amongst one’s immediate friends and family is now mediated through digital platforms carrying and distributing semi-private content in public fora. SM created opportunities for increased visibility, but have also raised privacy, ownership and censorship concerns (e.g., Gillespie, 2010): for marginalised or under-represented users, this digital storytelling can create opportunities for self-expression which come at a cost, and with attached labour (Vivienne & Burgess, 2013).

The everyday sharing of self SM allow also created swathes of ‘micro-celebrities,’ users with a public persona that audiences of fans or followers can consume, as the strategic use of intimacy appeals to whoever is watching (Marwick, 2015). This type of disclosure has now become the norm in the creator economy, where creators accumulate a following “through the textual and visual narration of their personal, everyday lives upon which paid advertorials – advertisements written in the form of editorial opinions – for products and services are premised” (Abidin, 2016:86).

Banet-Weiser observed the rise of “voyeurtainment” (2018: 51), or content creation and sharing through “technologies of the self” (p.56) enabling brand and audience management, self-promotion and advertising, broadcasting the flaws, mistakes and messiness of real life. This has normalised the sharing and commodification of private information, of presenting oneself through platformised authenticity and engaging in the relational labour (Baym, 2015) of building parasocial relationships with fans and followers. Such relational labour is, for Baym, the “ongoing, interactive, affective, material, and cognitive work of communicating with people over time to create structures that can support continued work” (2018: 19).

Being one’s authentic self online – and therefore “transparent, without artifice, open to others” even in one’s inner and private life (Banet-Weiser, 2018:61) - can be extremely lucrative for creators, who have normalised the sharing of personal experience through platforms, particularly for women (Duffy et al., 2022).

Creators may strictly control their SM content, presenting an upbeat, acceptable version of self, whilst simultaneously aiming for authenticity and attempting to maximise exposure to an audience – all critical tenets of the ‘influencer creep’ experienced by content creators and microcelebrities (Bishop, 2023). Such personal disclosure is inexorably tied to the platformisation of cultural production, whereby content creators depend on SM to produce and monetise content. These users become ultimately complicit in accepting platforms’ business model, governance and infrastructure that create dependency, and inequality” (Nieborg & Poell, 2018). This same platformisation that has so widely impacted creatives (Poell et al., 2022) now extends to everyday life, which increasingly must be shared and managed online to maintain personal and professional relationships and supplement increasing employment instability (McRobbie, 2015). Influencing practices have become the norm in the lives of creative industry workers, but “influencer creep” (Bishop, 2023) extends to ordinary users’, whose personal lives are increasingly visible online. As billions of users worldwide use SM to express themselves, work, network, curate and archive memories (Zhao et al., 2013), the possibility for involuntary context collision is always present (Davis & Jurgenson, 2014; Loh & Walsh, 2021). This creeping of influencer practices into everyday life can help us better understand colliding or colluding contexts, or the flattening of one’s audiences with the blending of different identities and viewers (Davis & Jurgenson, 2014; Duguay,^2016^; Vitak, 2012).

## Context collapse and the challenges of visibility

Much of the offline and online self-presentation literature (e.g., Duguay, 2016; Papacharissi, 2012) relies on Goffman’s, 1959 analysis of the structures of social situations. Using theatre as a metaphor, Goffman argues that the actions and image delivered and presented in public constitute a ‘front stage performance,’ on which an actor/person is judged. In this performance, an actor can explicitly share information, which is ‘given’. However, their innate characteristics and unconscious moves may cause for information to be ‘given off,’ or inferred by the audience. Goffman’s use of theatre in the description of self-presentation implies that we all present a performed version of ourselves to audiences, to generate certain impressions. We have come to accept that this is now a key part of crafting SM identities and of communicating through semi-public or public platforms that inevitably carry the risk of collapse such that identity performances are seen by the wrong audience.

Goffman’s metaphor takes new meaning in the age of SM, where our personal and professional lives are mediated through technologies that are not neutral, and that rely on opaquely made and governed algorithms to grant or deny success or visibility (Are, 2023). In this reality, virality and visibility tied to one’s profile may arise out of context, prejudicing creators’ reputation and work. Indeed, SM present a well-documented ‘remembrance’ risk: platforms act as an archive of every posted opinion or experience, which can help when remembering good memories, but also complicate self-presentation when past and present clash. Brandtzaeg and Lüders (2018) therefore talk of ‘time collapse’, particularly on platforms like Facebook, Twitter and Instagram, which require or allow users to post under their real name, connecting their online and offline selves. Communicating in public is therefore fraught with risk (Duffy & Hund, 2019) - not just of criticism, but also of hate and harassment, which often intersect with social identity, greatly affecting those more marginalised within the realms gender, sexuality, race, age, and class.

Studies on dirty work (Hughes, 1962; Slutskaya & Game, 2022) consistently show that stigma related to the wrong self-presentation or impression tend to hit marginalised communities harder, making them at once hypervisible – and therefore subject to harm and threats – and invisible when in need of help (Stegeman et al., 2024). Threats such as doxxing (or the digital publication of individuals’ identifying information), stalking and other online harms, as well as harassment tied to work identities have greatly affected creators such as sex workers, who often keep their work identity separate from their state identity to avoid discrimination and surveillance (ibid.).

Even users who seek to behave authentically on platforms must then engage in a form of “calculated authenticity” (Pooley, 2010), or a crafted version of themselves that is palatable to audiences but that does not alienate employers (Abidin, 2016) and that keeps them safe, requiring taxing emotional and administrative labour.

This creates challenges of “boundary management” (Kaul & Chaudri, 2018: 6), a set of calculated authenticity and self-tailored disclosure practices including “strategic acts of revelation and concealment, a delicate form of self-promotion and self-protection” akin to the activities of women in the public eye, such as political bloggers, activists, journalists, and academics who have come to anticipate networked hate (Duffy & Hund, 2019: 4997). In this sense, anyone posting content to be seen online must surveil themselves (Maddox, 2023) while simultaneously protecting themselves against practices like doxxing, which can trigger offline harm (Glatt, 2023). This is what Duguay (2022) calls “identity modulation,” or a continuous decision-making process about whether and how much particularly LGBTQIA+ individuals should make their sexual identity recognisable against personally identifying information (such as names, face-showing photos) on SM.

These challenges, which Glatt calls the ‘intimacy triple bind,’ also take a higher toll on marginalised creators, who need to keep sharing content to work in a precarious and hostile environment under capricious platform governance. Indeed, SM governance adds further strain to sharing oneself online, as users must constantly renegotiate one-to-one and public communications boundaries to avoid breaking platform rules (Ye et al., 2022), which essentially “monitor their normal social interactions” (Kaul & Chaudri, 2018: 6), while appeasing the algorithm to be seen (Duffy, 2020).

The stakes are therefore already very high for anyone posting on SM, either as a professional creator or as an individual whose life requires an online presence (Loh & Walsh, 2021). The situation, however, becomes more complex for users straddling different identities or, as Baym and boyd (2012) write, simultaneously navigating “different social and cultural worlds brought together in single social media sites” with multifaceted lives (p. 324).

Earlier work by Warner (2002) and Byron (2019:337) examined the importance of ‘counterpublics,’ where those who struggle to express themselves in larger or more public environment create “smaller publics with different sets of practices, membership, and ‘rules’”. The common practice of creating fake Instagram accounts (finstas) to allow greater authenticity with a smaller audience is an example of this (Dewar et al., 2019), although visibility control doesn’t necessarily require a fake account. If in these smaller spaces, people facing stigma can be themselves, but the collapsing of this context and the revelation of the stigmatised self to the larger public one could be problematic. These individuals – and especially LGBTQIA+ users and sex workers (Are et al., 2024; Duguay, 2022) - typically struggle in places where “default publicness” (or real-name policies) are the norm, and prefer fora where identity is more nuanced or, potentially, anonymous.

The fear of time collapse or context collision can create tensions when presenting oneself across audiences as an individual straddling different viewers, experiences, backgrounds (Vitak, 2012). Context collision can also have serious negative consequences for professionals, such as embarrassing or career-threatening repercussions over their SM posts, or merely problematic situations stemming from the merging of personal and professional audiences, making it a highly emotive issue, particularly for those at the early stages of their career (Loh & Walsh, 2021).

For Carr (2020), however, *context* is not SM’s main issue - *content* is, leading him to define *content* collapse as “the tendency of SM to blur traditional distinctions among once distinct types of information — distinctions of form, register, sense, and importance.” As SM become the main conduits of information ranging from direct messages to news, opinion and entertainment, this homogenises and trivialises disparate information, as well as how we respond to it. For Carr, this consolidates platforms’ power over our public and private lives, echoing Poell et al.’s (2022) platformisation. For this reason, we use Davis & Jurgenson’s (2014) distinction between context *collision* and *collusion*, given its focus on intentionality.

Our study therefore continues the trend of initial research efforts observing digital identity management (Duguay, 2016, 2022), particularly by those who post simultaneous work identities without sanitisation (Palumbo, 2024; Vitak, 2012).

## Multiple work identities and boundary management

The sharing of one’s personal identity – especially when this differs radically from a mainstream work identity – can result in a range of consequences for workers, but the risks grow when these multiple identities related to different and parallel forms of work.

Taking on additional ‘platform work’ may be driven by economic precarity (McRobbie, 2015), however holding different work identities can also be a way of busting taboos, opening minds, normalising professions and hobbies and, thus, facilitating social change. Indeed, holding multiple jobs can play a role in societal structural changes, including changes in economy, learning and re-training (Järvensivu, 2020). And, although people may take up additional work to improve their finances or curricula, leading some of them to keep this private to avoid looking unqualified, research found that employees cited monetizing a passion project (Caza et al., 2018) and seeking more autonomy through self-employment to align their work to their values (Abord de Chatillon et al., 2023) as reasons to take up different work, including work with a SM dimension.

For instance, while observing yoga teachers, Hufnagel and Spraul (2022) found that rather than setting out to teach, these workers were motivated by the additional meaning yoga gave to their lives. However, participants began feeling a conflict between their original work identity and their perceived new ‘yoga’ identity, craving more meaning in their professional lives as a result.

Some individuals take on a ‘second job’ or come to a job from a background that not only relies heavily on SM promotion, but also contains stigmatised elements such as nudity or sex work (SW) that may seem unpalatable to their colleagues. SW is a prime example of how a profession that requires considerable emotional and physical labour is often considered morally tainted, to the point that associating with it results in exclusion from other work opportunities (Morgillo et al., 2026). The term was coined by feminist sex worker activist Carol Leigh (1979) to unite those engaged in full-service SW, porn performers, dancers etc. and recognise the labour going into the sex industry, which had previously only been seen as a source of crime, exploitation or lacking morals even by the feminist movement.

SW provides relevant precedent to studies on multiple, platformised work identities because, as Bowen (2021) argues, sex workers (SWs) take on work available to them within precarious labour markets, meaning they may supplement incomes *with* SW or benefit from its flexibility, and they may not necessarily want to exit the profession for a ‘square job’.

Yet, SW has socially been linked with ‘dirt’ and connected with social exclusion, as social mores make SWs part of an ‘out group’ (Huges, 1962). Moral taint, which implicates ‘sinful’ actions or occupations, is often associated with dirt and dirty work, which can overlap with “stigmatized categories of socio-economic status, racioethnicity and gender” such as SWs (Slutskaya & Game, 2022:191). Taint is part of a process of social construction, influenced by historical and cultural circumstances and by politics, meaning that marginalisation and stigmatisation are often generated by liberal and neoliberal economics, from which the success of those who use SM to share their lives cannot be separated (ibid.).

SW is, crucially, also an over-policed yet potentially lucrative and accessible area of content production (Are & Briggs 2023), greatly affected by both platformisation and influencer creep in dealing with applications of moral taint, precarity and relational labour via digital content requiring management of different identities (Stegeman et al., 2024). Indeed, returning to Bishop’s tenets of influencer creep, we can see that those engaged in SW, particularly on platforms such as OnlyFans are required to promote their services in part through sharing aspects of identity to give clients a sense of authenticity, tasks which require a significant amount of emotional labour (Schuchmann, 2025). Creators like these may also collude their contexts, engaging in what Palumbo calls the “intentional blending of sexual content with mainstream or accepted elements to communicate targeted messages while avoiding platform restrictions or societal norms” (2024: 111).

It is in this multi-faceted context that we wish to understand the experiences of those that have shared work contexts *without* attempting to sanitise or change their self-presentation, to reinforce that SW *is* work and that SWs and those doing adjacent work are professionals in their own right. The experiences cited in this study show that while SWs face additional reputational challenges due to societal stigma, they provide great insight to adopting multiple roles more or less publicly in an increasingly platformised yet precarious economy. We therefore continue the conversation started by Palumbo’s (2024) “sexual content spectrum,” which captured the range of contexts, interpretations, and nuances in posting sexual content.

This paper centres on what Bowen (2021) calls ‘side hustles’: jobs carried out alongside another form of work. For some, but not all of our participants, these side hustles are SW or SW adjacent, meaning that context collusion carries specific risks for them. Nonetheless, our other participants faced different kinds of risk when going public with both forms of work. Our aim was to explore these different risks, but also understand the benefits to context collusion across distinctly different individual choices.

## Methods

We gathered data via five semi-structured, ethnographic interviews. We recruited participants through our SM, personal and professional networks, reaching out via direct messages on platforms and then subsequently via email, offering more information about the study and sharing a consent form. Recruitment focused on individuals who had publicly discussed the opportunities and challenges of colluding their content and contexts. As such, our sample is limited to what we were able to see in our network and not standardised according to specific backgrounds or identities, but simply driven by finding participants who had made the decision to go public with multiple and very distinct work identities.

To take part in the study, participants had to have joined their different identities and their respective audiences via their SM. Those who were successfully vetted and who met these inclusion criteria were paid £50 for their time after finishing the interview.

We found qualitative research interviews, consisting in researchers asking open-ended questions to collect rich data from participants, the best method to understand the nuances of the life experiences our questions would elicit given the topic of this paper (Roulston, 2022).

Our interviews were ethnographic, lasting no longer than one hour and conducted online, through the videoconferencing software Zoom, which allowed for recording and transcription, and which was participants’ preferred option to meet. Recordings were then deleted immediately after transcription, which was done manually through Microsoft Word documents later sent to participants to approve before including them in the study.

Interviews focused on participants’ description of spaces, actions or events on SM or in real world contexts. We chose this approach because of our own experiences of going public with two distinct working lives, which meant we could participate in the settings we were studying as is typical of ethnographic interviews. At the outset, we agreed to interview each other to understand our own experiences of context collusion – the first author as a pole dance performer, activist and academic with SM profiles joining all these identities, the second author as an academic professor and ceramicist with semi-collapsed SM profiles.

### Scoping interview

Starting out by asking each other to describe the work identities we straddled, our scoping self-interview highlighted the different ways in which our identities outside our conventional workplaces informed our day-to-day work.

Aside from providing access to considerable participant networks who may otherwise be reluctant to speak to ‘outsider’ researchers, The first author's pole dancing experience provided insights into lived experiences that informed their studies, while the second author's recent experiences as a ceramics student greatly aided their day job as a professor, allowing them to relate to students’ needs in the modern university and to explore material and digital content creation. The first author expressed the need to explore, perform and present their pole dancing identity as a necessary survival journey following traumatic domestic and sexual abuse experiences despite societal views of this being threatening to her emerging academic career, all the while honouring its roots as an art and a sport created by strippers. The second author is an established academic who has recently completed an undergraduate degree in glass and ceramics and now works at an independent ceramics studio. We perceived others’ understanding of our identities differently: the second author found colleagues dismissed their engagement with ceramics as a mere hobby, while the first found their experiences were often taken so seriously that they could be seen as a hindrance towards conventional employment.

The self-interviews raised issues we subsequently tackled in our interviews, in relation to managing different work personae individually, through SM and through HR departments, as well as the emotional and admin labour involved in maintaining them while remaining ‘professional’ in our day job.

### Participant stories

We established this study’s aim and sample following Malterud et al.’s (2016) work, which argues that studies prioritising similar experiences do not require a high number of participants, as the data featured will be rich and relevant. We chose to present their stories as case studies, since they are unique and diverse, reflecting their own specific way of coping with different contexts. They can therefore provide a starting point beyond the involuntary context collapse vs. persona sanitisation binary, and benefit the field with more nuanced reflections.

Our participants were: Madelaine, a dominatrix and tech CEO; Fiona, a chemical engineer and published novelist; Peachy, a pole dancing doctor; Whoretographer, a photographer and sex worker and Matt, an academic and puppeteer. As is visible from this eclectic mix of people from different occupations and backgrounds, a key limitation of this study is that we cannot generalise these experiences according to a single profession.

We summarise our participants’ unique experiences below, giving information around gender, sexuality and race as these impacted some of the decisions made by our participants when managing their SM identities. Again, we emphasise that such information offers a more complete picture of the individual, but is not shared with the intention of suggesting any generalisability of the associated data and practices. We also recognise the need for caution with our own and readers’ understanding of participants’ stories, recognising the dangers of unconscious bias processes that may inadvertently affect interpretation.

#### Madelaine

Madelaine is a cisgender white woman in her thirties who describes herself as a CEO in the sex and ethical tech field and as a successful dominatrix. When she founded her tech company inspired by her experiences in SW, she joined the two identities because she was tired of the admin and emotional labour required by juggling two different personae.

When speaking publicly about her work, Madelaine initially created a ‘fake’ real name for her dominatrix persona, to separate her real identity from the shame, stigma and financial discrimination that target SWs. This aided her family life, during which she sometimes worried of being recognised in front of her children, a teenager and a toddler.

Having already been targeted with stigma under her fake name, and following the heartbreak of having had to publish research and work with a name that was not hers, Madelaine felt her separate identity only gave her a false sense of protection. Recognising the privilege of having a supportive family, Madelaine approached her closest relations to discuss the risks and opportunities of joining her identities.

Tired of having to check herself when speaking about different types of work or being approached for projects, and to normalise SW publicly in the same ways as she did in her private and family life, Madelaine came out with her real name. She now finds she has saved herself a lot of admin and fear that one identity could affect the other.

#### Fiona

Fiona is a cisgender white woman and chemical engineer with over 40 years of industrial experience, during which she held senior management positions in a multi-national company. She is also a successful crime fiction writer, with seven published novels. She initially began writing whilst working in an engineering environment, where she had a challenging relationship with a former boss and found writing to be a form of release. She would get up at 5am and write until her professional workday started, occasionally returning to writing in the evenings.

Initially she kept her new writing identity separate from her engineering workplace, recognising that her boss and some of her high-profile international clients would not necessarily approve. She publishes under her married name, whereas she kept her maiden name in the engineering workplace and was careful to limit her use of SM to professional platforms such as LinkedIn. Ultimately, however, she recognised that the SM activity tied to being a published author did not sit well with such secrecy and decided to open up at work about her writing career, letting her HR team know her writing was done entirely in her spare time.

Following a series of talks associated with her writing, Fiona was offered a post in higher education, teaching health and safety to a multi-racial student body. Again, she decided to remain open about her writing, although she does not deliberately promote her books to her students, partly because she is aware that exposure to some of the more explicit sex scenes in her writing could prove awkward. Fiona enjoyed having a female protagonist who had agency in choosing who she would sleep with, describing this as her “revenge by having plenty of beautiful, disposable men,” in similar fashion to conventional male protagonists.

Since cross-posting on different SM platforms, she now worries that any push-back she may get from her audience will be from readers who take issue with her associations with the Middle-Eastern oil industry and with the petrochemical industry’s climate change implications. She therefore tries to use both of her identities to make a reasoned case for sustainable action.

#### Peachy

Peachy is a British-Asian, cisgender doctor in her late twenties working towards specialisation and a pole dancer who teaches workshops, performs, judges at pole dance competitions and creates viral online content. She took up pole dancing at university, when she joined a pole society and fell in love with pole to balance a degree she found overwhelming.

Initially, she felt she had to keep her passion separate from her everyday life. After attending a talk about professionalism before graduating and anonymously asking a question about continuing her medical career while openly pole dancing, Peachy was told unequivocally that the two identities could not collide.

Disheartened by this response and adamant there was no harm in pole dancing, Peachy started researching others who successfully blended pole dance with a professional career. She found several examples of people who did so - she referred to another pole dancing doctor and to The first author - and, while weighing up the pros and cons of blending her identities, she started posting more openly on her pole dance account. Once she found more courage in numbers, she became fully open about her life to avoid having to manage the stress of keeping her lives separate.

As her following grew, Peachy initially struggled to manage her workload, focusing on night shifts to be able to take up pole opportunities during the day. Exhausted, she has now taken a step back after finishing her training to work locum shifts and have more flexibility.

Her workplace raised further challenges when one of her medical directors found some of her videos following formal complaints that a pole dancer could be “a disgrace to the NHS,” and that she could even be breaking patient confidentiality due to the appearance of a corner of a hospital in her video. This resulted in an investigation that Peachy found traumatising, but that ultimately cleared her because the hospital was not identifiable. Nonetheless, Peachy found research showing that investigations of this kind affect women, and women of colour, more often.

Additionally, more senior male colleagues engaging with her content made her feel watched, resulting in having to further manage her online presence to avoid them.

Still, to this day Peachy is proud of having blended her identities for the normalisation effect this can have on people going through similar challenges, and for the opportunities and communities she found on the back of it.

#### Whoretographer

Whoretographer is a white, cisgender woman, as the participant changed their understanding of their identity during the proof process queer photographic researcher and artist in her early twenties. She uses use photography and image-making to explore and understand SW, gender performance, censorship and labour rights, an approach rooted in her experience of and connections within the sex industry.

She adopted this approach in her second week at university, when her partner was a SW part of a socialist SWs cooperative running their own independent events. She was asked to photograph these events at the start of their degree, resulting in her profile in the industry growing.

While initially she was using her legal name for their university work, she began using a pseudonym once her research and photography work started aligning with her outside interests. This felt necessary when engaging in commercial photography opportunities, where SW would be stigmatised. However, increasingly, after being known for being the photographer at kink, sex party and SW cabaret events, she began linking her accounts more and showing her face under Whoretographer, also to ensure that the people she was photographing felt safer when being shot by an insider.

Having become more open about her work, Whoretographer found some artists and lecturers did not ‘get it’ or found space to platform it. Other times, she faced harassment from lecturers who would suddenly tell her about their sex lives.

Although she did not feel supported by lecturers or students, having already experienced financial, digital and societal discrimination meant Whoretographer is used to stigma, allowing them to join her identities. This, ultimately, was important for her not just to show she were was a safe photographer at events, but also to challenge the pervasive stigma surrounding SW, to highlight the validity of artistic work of a sexual nature and to teach the next generation of photographer about sex, consent and gender rights.

#### Matt

Matt is a white, gay cisgender man working as an associate professor in a computer science department, where he teaches and researches topics linked to human-computer interaction and human-centred artificial intelligence. He is also a professional puppeteer, working for a company that organises large-scale puppetry performances. Matt had always “been obsessed” with puppets as a child, but he studied Psychology at University and was drawn into the field of HCI for his PhD. Before taking up a faculty position, he also worked in a university professional services department.

Matt found puppetry through his partner, who ran a puppet festival and asked him to be involved. His initial work related to logistics and artist liaison, but subsequently, he and his partner began creating puppetry together and now, across all his SM platforms, he alternates the use of the joint label ‘academic and puppeteer’ or ‘puppeteer and academic’.

Whilst being open about both professions, Matt has noticed that academic colleagues are interested in his puppetry, but they will sometimes be dismissive in assuming it is a hobby rather than a profession. On the other hand, those from outside academia tend not to take an interest in his academic work, sometimes dismissing him as a ‘programmer’ as opposed to someone who researches community participation in the development of new technologies. He has tried to be more transparent about these two identities, exploring the idea of using puppetry in his academic work. However he now feels he has an established and equally important identity in both worlds, and he can legitimately claim he is both a puppeteer and academic.

### Analysis

After transcription, we analysed interview data with a thematic analysis (TA), which we present as a combination of our analysis with participants’ answer excerpts. TA is a qualitative method ideal to identify, analyse and report themes or patterns within a study’s data (Braun & Clarke, 2006).

Themes originate from an intersection of the data with researchers’ theoretical assumptions, analytic resources and skill (Braun & Clarke, 2019). We developed these through coding, which we conducted following a pre-selection of relevant quotes and concepts into temporary categories via an Excel spreadsheet, followed by a coding meeting where we settled on three overarching themes, presented below:

1. Celebrating a dual life;
2. Stigma and reprisals;
3. Managing workload.

### Celebrating a dual life

Across the board, our participants had reached a point where they refused to ‘choose’ between a sanitised professional identity (academic, doctor, chemical engineer, CEO, photographer) and a more creative, attractive or stigmatised additional work identity (novelist, puppeteer, SW). For them, both had equal if different value, be it monetary, ethical or creative, sparking different feelings and awakening different parts of themselves.

But there were notable differences between participants. In particular, some had entered their second profession as a hobby, but later realised that it reflected more of their ‘true identity’ than their more conventional job, and therefore sought opportunities to monetise their passion, reflecting a search for greater meaningfulness at work (Hufnagel & Spraul, 2022; Järvensivu, 2020). This meant the hobby became a second profession, but one that they felt had equal status to their more traditional job. However, those participants who had experiences of SW ***before*** entering another profession had to make different decisions and give careful consideration about how and whether to blend their two lives. Thus, whilst we see a similar concern for content curation across all participants, we did observe that those participants who commercialised their former hobby were allowed more ‘play’ and faced less risks than SWs.

All participants engaged not just in *context*, but in *content* collusion (cf Carr, 2020), which meant that, as spaces that integrated several work and life dimensions, SM did not only require users to share different parts of selves (Poell, 2020): they intuitively felt like the right spaces to celebrate content from both lives, the duality of which they embraced not just out of personal need, but as a principle. For example, pole dancing doctor Peachy rejected the prevailing idea that she should stifle her love for and sharing of pole dancing to conform to a “*cookie-cutter culture’*,*”* adding that “*there’s nothing wrong with being someone else outside of work*,” while Matt stressed the importance of being both an academic and puppeteer as equally valid professions:“[My academic and puppeteer identities] are both professional identities, which is probably why I put them both on my social media profile because they are equal to me”.

Other participants publicly acknowledged their two selves, but kept some of their social media content separate (e.g. acknowledging SW identity on their public ‘colluded’ identity SM, but only posting their porn on channels that allowed it, or discussing how their professional expertise lended them credibility in their creative endeavours, without sharing this creativity on their purely professional channels). Their desire to be upfront about their different selves and their lives in and outside of work triggered meaningful reflections around the value of such authenticity:

“My mum, my sisters, we’ve always said, ‘If there’s nothing to hide, then there’s nothing to find.’ So when it comes to introducing myself to my friends or their friends, it’s always like, ‘Oh, what do you do?’ ‘Oh, well, it’s a bit interesting actually. I do this,’ and I always am very upfront about what I do” (Madelaine, dominatrix and tech CEO).

“Because your work expects you to behave in a certain way, there’s a stiff upper lip kind of attitude where you just keep your personal life outside. And actually I think it’s much healthier […], a much pleasanter situation where you feel you can say, look, I’ve got to take my kid to the dentist and you don’t feel like the whole office is kind of looking at you like you’re a slacker.” (Fiona, chemical engineer and novelist).

Across the board, our participants recognised that they were somewhat unusual in collapsing their identities, but they also hoped to act as role models in challenging prevailing views about what is ‘appropriate’ in the workplace, about what constitutes a ‘profession’ or in tackling misconceptions of certain types of work, and even in supplementing one type of work with knowledge from their other activities.

“The tension then, is that it’s like, ‘Oh, it’s your little hobby that you do.’ And it’s like, well, no, actually […], I have work professionally as a puppeteer. I might not be at work work, but it is still work” (Matt).

“I’m trying to destigmatize normalcy in sex work spaces and kind of go: we don’t lounge around in lingerie, that’s a falsified idea and you’ve built that in your head” (Madelaine).

Our participants strongly demonstrate the value of *both* traditional and alternative careers by actively building synergy between them. Similarly to our own experiences, participants utilised knowledge gained through their creative and/or stigmatised practice in their more conventional work identities and jobs, which were enriched and not hindered by their less conventional identities. Platforms seemed to have played a positive role in this, aiding participants in seeing there are other people straddling multiple lives, which are made visible by SM’s blurring of distinctions amongst types of information (Carr, 2020).

In line with previous work (Nieborg & Poell, 2018), participants were therefore both complicit *and* interested in harnessing the opportunities of platformisation. However, the side hustles that platforms made visible to them often arose in situations of precarity (Bowen, 2021; McRobbie, 2015), and this visibility created the added difficulty of fitting nuanced human beings with private bonds into a public and professionally acceptable social media niche. Because of this, participants were also conscious of the affordances of the spaces they shared their lives in, carefully calibrating which content could fit in which context to avoid anticlimactic or inappropriate collusions – particularly in the case of prejudices against outside-work identities, or more sex-adjacent content and labour, again, reinforcing the notion that participants with SW adjacent content faced higher risks.

### Stigma and reprisals

Our participants struggled to deal with certain public or professional preconceptions, some associated with the stigma attached to sex-adjacent work and some associated with the idea that they were spending too long on what was simply a ‘hobby’.

Participants whose content had a sexual element faced different forms of online and offline harassment after collapsing audiences, leading to extra emotional labour to protect themselves while maintaining the ‘authentic’, sexy and available persona required by online SW (Schuchmann, 2025). Their desire for transparency would backfire if others chose to weaponise this knowledge or to make inappropriate assumptions.

“Lecturers will be very open with you about their personal sex lives. Maybe not in the most appropriate way. [.] And it’s a little bit like, OK, well, I can’t really go to HR or someone about this because I’m also sharing very personal, explicit things… [but] this is professional research and not the same as you telling me about the affair you had with it with a dominatrix in your 20s.” (Whoretographer, photographic researcher and SW).

“I’ve had really creepy encounters with men who are more senior than me, like consultant level, who are my supervisors, saying really inappropriate comments to me”. (Peachy).

“People will shout [my dominatrix] name from the car, and I have to ignore because or just kind of go, ‘Oh, wonder who they’re talking about,’ because I do not want my children to know that that’s normal, ‘cause it’s not. You know, it certainly shouldn’t be normal for them.” (Madelaine).

Our participants had to learn new techniques to navigate these hostile situations, which, many of them argued, would have been present anyway in case they were recognised without having purposefully joined their identities. Indeed, with SW and adjacent labour being viewed as ‘dirty work’ (Hughes, 1962; Slutskaya & Game, F2022), their lives and identities were not just seen as morally tainted, but also as professionally illegitimate or unworthy because they did not conform to perceived professional standards, making being ‘found out’ always a risk - a risk participants were tackling head on. This affected them personally and professionally, resulting in important reflections surrounding moral taint and its connection with perceived lack of professionalism or reputational challenges (Morgillo et al., 2026), which they did not see reflected in their day-to-day activities or identities.

“Whether you’re denied a bank loan or your PayPal account is shut down, or it’s a man at university, kind of questioning your legitimacy. It’s all the thinly veiled the same thing. So once you kind of learn how to navigate one of those, like, perhaps the kind of slight conversational demeaning stuff becomes a little bit easier” (Whoretographer).

“There is so much weight to put on the, ‘Oh, people can’t find out who you really are.’ But then I think to myself, ‘But why? What am I? What am I doing that is so wrong that it’s going to have people hunting me down?’ And if somebody is going to hunt me down, that’s a them problem, it’s not my problem. I shouldn’t be the one that’s hiding myself because somebody may do something” (Madelaine).

Still, harassment and trauma associated with ‘dirty work’ were ultimately made worse by the fact that haters had a new strategy – using their more traditional professional affiliations as a means of attack, as was the case when Peachy came under investigation in her traditional workplace.

Further, public opinions about SW meant that when engaging in content collapse or collusion, participants like Madelaine had to bear not just their experiences in mind, but their families’ too, adding another layer of labour to the process because of the need to protect her children:

“They understand that sometimes people might be mean to them about mummy’s job, and sometimes people might misunderstand me and my job and they might show them things that Mummy does for her job, but that you’re not to look at them. And then that brings out all sorts of questions like, ‘Well, can you show me?’ And it’s just the curt, ‘Sweetheart. No, I can’t.’” (Madelaine).

These participants, despite striving to be transparent, had to adopt a significant additional level of careful calculation, identity modulation and self-surveillance to navigate societal and platform norms (Duguay, 2022; Maddox, 2023; Poole, 2010). Their context collusion was more like Palumbo’s (2024) sexual content spectrum, blending different aspects of one’s life online to manage stigma and navigate the effects of visibility, rather than a completely unfiltered self-presentation. This spectrum seems to be an added requirement of platformisation’s blending of different life fields (Poell, 2020).

Moving away from highly stigmatised work, we found that our other participants also dealt with different forms of prejudice, largely around the preconception that a ‘dual’ life was an excuse for ‘slacking’ and that the alternative work would inevitably be detrimental to the ‘proper job’.

“When I when I was offered the job, I did go to HR … I didn’t want them to find out about it and think that I wasn’t doing my job… I deliberately didn’t ever mention [my writing career] in the interview because I think that just gives people a reason for thinking you’ve got other priorities? … And I was very careful that my day job was my day job and my writing time was done in my own time…” (Fiona).

We can understand from this that some participants (e.g. Peachy, Fiona) had visible roles in traditional workplaces that were relatively unforgiving in terms of any possible breach of conduct. For both of these participants, the risks of promoting a second identity were relatively high but negotiated in very different ways: Fiona with a desire to meet with her HR department in order to give them assurances of working ‘full time’ and showing total commitment to the job, Peachy in terms of managing the stigma associated with pole dancing in a medical profession that emphasised impeccable ethical conduct.

Strikingly, the onus of managing harassment, inappropriate comments or prejudices about their work abilities and/or commitment to the job was always put on our participants, and not on their colleagues or employers. In the situations they described, workplaces, colleagues, even academic institutions with safe-guarding obligations *were simply not equipped* for people’s ‘outside lives’, additional jobs or backgrounds, even though these have become increasingly common, if not required, in the SM age (Bishop, 2023). This inevitably led to additional labour, of what Peachy called ‘testing the waters’ in what to post and reveal, because their employers provided insufficient guidance, or were inconsistent in their attitudes towards ‘risky’ content.

“Everyone wants a radical and disruptor, and they praise you for it. But as soon as it becomes, like, tangible things that affect them or their part of it, then it’s like, oh, it’s a bit much, so a lot of it feels like people are very supportive when it makes sense to them. But as soon as it becomes a bit too fringe or a bit too extreme, they’re not” (Whoretographer).

Ultimately, participants would find themselves caught up in a double-bind: they found community, validation and support for their two identities, and like-minded people found the context collusion inspiring and even trail-blazing. Yet they also had to deal with HR systems that, despite a rhetoric of support for work-life balance, could not support work-work balance. In this voyeurtainment (Banet-Weiser, 2018) without benefits enabled by platforms that create additional work and opportunities but do not absorb the challenges they create, the relational labour created by collapsing one’s content seemed to be performed by participants alone (Baym, 2015, 2018). Platformisation in an increasingly precarious job market demands authenticity, but when people attempt to be authentic by showing their whole selves, they face several challenges to their safety, job and connections, mirroring other aspects of SM governance that are not kind to marginalised creators (Are & Briggs, 2023).

### Managing workload

The demands of our participants’ SM work came on top of the fight to find the time to create successful content. They all recognised that writing, performing, developing and promoting online content with limited time was in itself problematic, particularly in conjunction with other, more traditional work that was taken more seriously. Yet they were keen to emphasise the importance of creative content, for which they fought.

“I love performing and so at the moment I took a step back from my full-time medical career to explore that split a bit more, and because I was trying to keep it up during working full time and I was managing, but only just. Because I often had to like, sacrifice sleep to be able to do it.” (Peachy).

“I’m generally up at 5 and when I had to go into work, I would write from sort of half 5 till 7.30 and then shower and go to work. I’m not good at writing in the evening, but I will often do social media in the evening because it doesn’t take quite so much concentration.” (Fiona).

All participants described the difficulty of juggling different personae, both before and after deciding to blend them. This reflects the nuances of ‘face-work’, where the struggle to maintain a consistent self-image or ‘face’ in different contexts can be highly demanding ( Goffman’s, 1959 ). Some recognised that having a consolidated identity made audience management simpler, particularly when posting as an individual as opposed to an organisation.

“I think it is strategically sensible to do it and kind of just consolidate your social media presence of just like you, the individual… it makes less work, I think. Maybe it is complicated because you’re speaking to a kind of dual audience, but then I don’t think about that too much” (Matt).

Others struggled to consolidate an identity across different social media platforms, making additional work for themselves in the process of developing content streams for different platforms. A further complication arose with job change. For one participant in particular (Fiona), the move from a petrochemical giant into an academic post with more autonomy meant that she became less concerned with HR expectations associated with her primary role, and much more concerned with possible value misalignment, should her readers and new students question her former involvement with the petrochemical industry. In both cases, she decided not to ‘hide’ her work, but the stresses associated with this open practice changed considerably over time and were confounded by the need to operate across multiple platforms.

“I am finding it harder now that there are so many different platforms. […] So at some point I will have some kind of strategy where I decide what I’m going to use them for or stop using some altogether.” (Fiona).

Participants with sex-adjacent or SW identities found that managing stigmatised content could be particularly challenging, not least because some of them would take on an additional task: checking their audience to ensure that they and other members of their community would remain safe whilst also managing the ever-present threat of de-platforming (Are & Briggs, 2023). “*I regularly check through who’s viewing my stories. I’m quite like protective of my community*,” said Peachy.

It is perhaps surprising that in the face of these layers of additional work, participants remained convinced of the benefits of identity collusion, possibly because of how it would help to reduce content and context collusion anxiety in the longer term. Yet, their investment in their passion and the community backing they received seemed to make it worthwhile. Indeed, participants often stated that salve would come from like-minded communities, who stepped with the support that HR and colleagues failed to provide and who invested in their stories through engagement, purchases and conversation. Across the board, our participants said they would not have it any other way because, like Hufnagel & Spraul’s yoga teachers (2022), their less conventional identities often aligned with their values and informed the trajectory of their careers.

## Conclusions

Through an eclectic mix of case studies, we explored how people with apparently clashing work identities, including stigmatised ones, willingly collude their contexts and content. The means of collusion were somewhat unique to each of our participants, with some simply promoting two distinct identities without reservation and with others (such as Fiona) using elements from one profession to enhance her skills in the other. With these specific cases, we make a step towards examining context collusion *outside* identity sanitisation scenarios, or in the aftermath of accidental or unwanted context collusion, addressing a gap in platformisation, digitally-mediated work, labour and content creation studies.

What we found seems to be a snapshot of a moment in time for generations of digital natives such as Millennials and Gen Z, who progressively used technology to share their lives online (Vivienne & Burgess, 2013). This moment in time highlights not just the challenges of platformisation for individual workers (Poell et al., 2022), but a disconnect between traditional work processes and the experiences of those adopting these now not-so-new modes of work.

We found that participants who could make the most out of colluding their contexts also added a form of ***content*** collusion to their self-presentation (Davis & Jurgenson, 2014). Certainly, those who blended their identities successfully could find a way to justify how their secondary activity or stigmatised background aided their career path. Those who performed jobs that did not match did not feel the need to justify their activities or identities. Instead of stigma and prejudice against their job or identity, these participants largely grappled with the fear that their outside-work identity may be seen as ‘just a hobby’ and not taken seriously, or that it would be seen to take away from their day job.

Participants often chose to make their content collude because of their personal journeys rather than of a particular strategy. In line with Brandtzaeg & Lüders’s (2018) idea of time collapse, participants may have begun sharing parts of themselves on platforms *ahead of* realising how much of a central role these jobs or activities would have played in their lives, and then found themselves having to consider whether both could be accepted as a whole. Similarly to Hufnagel & Spraul’s yoga teachers (2022), their circumstances – finding motivation, inspiration, value and meaning through their outside work activities – led them not just to pursue said activities further, but to publicly make them part of their digital identities.

Further, participants who engaged in a job *previously to* the activity they associate more strongly with their career shared a combination of staying true to themselves, reducing their admin load and the stress of being ‘found out’ while acknowledging the learnings said job brought them to collude their contexts. Their stories show that identity management on SM is not black and white, and that users may inhabit grey areas that allow them more freedom, creativity and balance of personal and professional interests.

Chances are our participants may have still engaged in hobbies or attempted to enter specific professions even if SM did not exist. However, SM and the creator economy created lower barriers of entry into creative and content production industries (Glatt, 2023), removing intermediaries such as production companies and allowing people from under-represented groups or stigmatised backgrounds to engage in content production and become established in their field. By creating environments where different timelines and content – and thus pieces of self – could be displayed, SM therefore enabled participants to consider the benefits, and not just the drawbacks, of context collusion: as a result, the ability to *share* these activities online, to *connect* with others through them, promote their work and *expand* their opportunities via their mediated digital identities made context collapse useful and desirable for them.

Mostly, our participants’ stories provide reflections on how what Bowen (2021) calls ‘side hustles’ can be crucial, not just to supplement precarious or lacking incomes in a capitalist society, but also to seek meaning outside the traditional workplace (Hufnagel & Spraul, 2022). This leads us to conclude that many workplaces still have outdated expectations and processes when it comes to reputation, worker self-presentation and support towards improving their work life. Indeed, ‘content collusion’ (Carr, 2020) was not always a straightforward choice for participants, who recognised the work still involved a great deal of identity management, given different platform affordances. Essentially, SM came to represent different parts of their life, turning into the ‘places’ where they go for specific activities instead of merely being communication intermediaries. This reflects the increased platformisation of our lives (Poell et al., 2022), where everyday actions are increasingly mediated through digital platforms, but also reminds us of the risks involved in making decisions about which platforms to use, and how (Are et al., 2024). Similarly to previous work highlighting the precarity surrounding side hustles and platform work (Bowen, 2021; McRobbie, 2015), it was clear that, similarly to offline HR departments, platforms created opportunities but didn’t protect workers when related challenges arose. Further, although platformisation seems to demand authenticity (Banet-Weiser, 2018), this authenticity created risks of exposure *and* of flattening complex, nuanced selves into a box that an algorithm could recommend.

As a result, in absence of support from the tech companies that have created these spaces, most workplaces would benefit from training their staff about the different constraints and opportunities provided by the SM age, and recognise the benefits and/or needs of side hustles more conventionally.

Finally, given our own experiences of different levels of content and context collusion, we note not just how little support institutions provide in the navigation of these not-so-new identity management challenges, but also how unique and network-dependent these choices can be. For the first author (who notes some parallels with those participants using nudity in their work) the journey was gradual and critically dependent upon career stage and upon the likely impact of full disclosure on their future research career. For the second author, the opportunity to move into ceramics was dependent upon the ability to go part-time in the more traditional workplace and reflected a need to move away from too much ‘screen time’. Ironically, she was somewhat surprised to find that SM promotion was expected in the new role too. This brings us back to how influencer creep seeps into many different aspects of professional life (Bishop, 2023), but while other aspects of digital work - e.g., videoconferencing, digital portfolios, instant messaging - have been normalised and widely encouraged by workplaces, content creation remains a more opaquely and confusingly adopted practice, touted as a benefit when aligned to a work identity, but viewed as a challenge otherwise.

We hope that some of the stories shared by our participants can help not just extend an already developing research field, but also provide inspiration to those grappling with similar challenges.

## Data Availability

The data that support the findings of this study are available on request from the corresponding author, further information is available at: Are, Carolina; Briggs, Pamela (2025). From context collapse to context collusion: new approaches to social media identity management. Northumbria University. Dataset. https://doi.org/10.25398/rd.northumbria.29998387. The data are not publicly available to preserve the privacy of research participants, who were selective about which information about them we could disclose given the nature of their work and public identities.
